# Severe Traumatic Brain Injury Requiring Surgical Decompression in the Young Adult: Factors Influencing Morbidity and Mortality – A Retrospective Analysis

**DOI:** 10.7759/cureus.3042

**Published:** 2018-07-24

**Authors:** John Ogunlade, Chris Elia, Jason Duong, Paulino J Yanez, Fanglong Dong, Margaret R Wacker, Rosalinda Menoni, Todd Goldenberg, Dan E Miulli

**Affiliations:** 1 Neurosurgery, Riverside University Health System Medical Center, Riverside, USA; 2 Neurosurgery, Riverside University Health System Medical Center, Rancho Cucamonga, USA; 3 Clinical Research, Harvard University, Boston, USA; 4 Clinical Research, Western University of Health Sciences, Pomona, USA; 5 Neurosurgery, Arrowhead, Moreno Valley, USA; 6 Neurosurgery, Arrowhead, Colton, USA; 7 Neurosurgery, Kaiser Permanente Fontana Medical Center, Fontana, USA; 8 Neurosurgery, Riverside University Health System Medical Center, Moreno Valley, USA

**Keywords:** severe traumatic brain injury, traumatic brain injury, decompressive craniectomy, craniotomy, neurotrauma, glasgow outcome scale, glasgow coma scale

## Abstract

Introduction: Severe traumatic brain injury (TBI) is a leading cause of morbidity and mortality among young adults. The clinical outcome may also be difficult to predict. We aim to identify the factors predictive of favorable and unfavorable clinical outcomes for youthful patients with severe TBI who have the option of surgical craniotomy or surgical craniectomy.

Methods: A retrospective review at a single Level II trauma center was conducted, identifying patients aged 18 to 30 years with isolated severe TBI with a mass-occupying lesion requiring emergent (< 6 hours from time of arrival) surgical decompression. Glasgow Coma Scale (GCS) score on arrival, type of surgery performed, mechanism of injury, length of hospital stay, Glasgow Outcome Score (GOS), mortality, and radiographic findings were recorded. A favorable outcome was a GOS of four or five at 30 days post operation, while an unfavorable outcome was GOS of 1 to 3.

Results: Fifty patients were included in the final analysis. Closed head injuries (skull and dura intact), effacement of basal cisterns, disproportional midline shift (MLS), and GCS 3-5 on arrival all correlated with statistically significant higher rate of mortality and poor 30-day functional outcome. All mortalities (6/50 patients) were positive for each of these findings.

Conclusions: Closed head injuries, the presenting GCS 3-5, the presence of MLS disproportional to the space occupying lesion (SOL), and effacement of basal cisterns on the initial computed tomography of the head all correlated with unfavorable 30-day outcome. Future prospective studies investigating a larger cohort may provide further insight into patients suffering from severe TBI.

## Introduction

Traumatic brain injury (TBI) is a life-changing event that affects the patients, families, and the community at large. In 2013, an estimated 2.8 million people suffered from TBI in the United States [1]. Of those, 282,000 people were hospitalized, and approximately 50,000 people died of TBI [1]. Among patients with severe TBI (i.e., with a Glasgow Coma Scale [GCS] score ranging from 3 to 8), 60% die or survive with severe disability requiring 24-hour care [2-3]. Following the primary traumatic insult, these severe TBI patients require aggressive medical and often surgical treatment to prevent secondary brain injury [4-6]. Demographics, cerebral hemodynamics, serum biomarkers, and surgical approaches (e.g., craniotomy or craniectomy) have all been investigated to assess prognosis after severe TBI [7-14]. Previous studies focus on the influence of early decompressive craniectomies on outcomes in the setting of severe TBI with refractory intracranial hypertension [7, 15]. Although many studies have found correlations pertaining to favorable or unfavorable outcomes, this remains a debated topic.

Therapy in this patient population is often directed towards the prevention of malignant intracranial hypertension [9, 11]. Although there are a growing number of studies assessing the benefit of craniectomy in severe TBI, the data are limited concerning factors predictive of overall outcome in young adults [7, 15-17]. Moreover, old age has been associated as a poor prognostic factor in patients with severe TBI [8]. We believe that a study investigating morbidity and mortality of severe TBI selected for craniotomy or craniectomy in young adults without associated comorbid conditions may provide more insight into the intrinsic disease process. This current study aims to achieve a better understanding of the morbidity and mortality associated with severe TBI in the young adult population (i.e., patients aged 18 to 30 years) requiring emergent neurosurgical intervention upon presentation.

## Materials and methods

We conducted a retrospective review from 2005 to 2015 at a single Level II trauma center identifying all patients with isolated severe TBI requiring emergent (<6 hours upon presentation) decompressive craniotomy or craniectomy for a space-occupying lesion. Severe TBI was defined as a presenting GCS score of 3 to 8 after resuscitation.

Patients included in the study were 18 to 30 years of age with isolated severe TBI requiring emergent neurosurgical intervention within six hours of arrival. All patients had at least 30 days of follow-up. Patients were excluded if they had a concomitant spinal cord injury, other significant traumatic injuries, or if they presented as a GCS of 3 with absent brainstem reflexes and bilateral dilated non-reactive pupils at the time of arrival. Patients not requiring emergent surgical decompression upon arrival were not included in the study. Patients with epidural hematomas as the primary space-occupying lesion without underlying parenchymal injury were excluded. Patients were also excluded if confounding variables (e.g., drug intoxication, medication overdose, or significant pre-existing co-morbidities) precluded an accurate assessment of their GCS on arrival or further complicated their post-operative management (e.g. autoimmune disorder, seizure disorder, coagulopathy). The decision of craniotomy or craniectomy was made by the primary operating neurosurgeon. All craniectomies were at least 10 cm x 15 cm. This study was approved by the institutional review board committee.

After identifying the study population (n = 50), we collected the following independent variables for statistical analysis: age, gender, injury sustained, degree of mid-line shift, effacement of basal cisterns, surgery performed, presenting GCS score, GCS score at time of discharge, length of hospital stay, time to follow-up, surgical complications, and Glasgow Outcome Score (GOS) 30 days after surgery. Disproportional midline shift (MLS) is defined as MLS greater than the diameter of the space occupying lesion (SOL). Closed head injury (CHI) is defined as TBI in which the skull and dura are intact. Depressed skull fractures (DSF) in this manuscript are defined as TBI in the setting of an open skull fracture or any other skull fracture with associated dural injury. In this study, a favorable outcome was defined as a GOS of four or five at 30 days from the day of surgery. An unfavorable outcome was defined as a GOS of 1 to 3 at 30 days from the day of surgery.

All statistical analyses were conducted using the SAS software for Windows version 9.3 (Cary, North Carolina, USA). Descriptive statistics were presented as means and standard deviations for continuous variables (e.g., age), along with frequencies and proportions for categorical variables (e.g., gender). Chi-square statistics were conducted to identify the outcomes associated with the two surgical procedures (craniotomy or craniectomy). The Wilcoxon rank sum test was conducted to identify whether the median length of stay was different between patients receiving craniotomy or craniectomy. We conducted a logistic regression analysis to identify the factors associated with unfavorable clinical outcomes, defined as a GOS from 1 to 3. All statistical analyses were two-sided. A P-value < 0.05 was considered statistically significant.

## Results

Fifty patients with severe TBI were included in the final analysis; 86% were males with an average age of 22.38 ± 3.12 years. Twenty-four (48%) patients underwent a craniotomy and 26 (52%) underwent a craniectomy. Most patients suffered a closed-head injury (80%), followed by depressed skull fractures (DSF) (16%), and gunshot wound to the head (4%). All patients had at least one space occupying intracranial hemorrhage of subdural hematoma or intracranial hemorrhage. Fifty-four percent were a GCS of 6 to 8 upon arrival (Table 1).

**Table 1:** Patient demographic information (N = 50) Abbreviations: CNS, central nervous system; CSF, cerebrospinal fluid; GCS, Glasgow Coma Scale; RTO, return to operating room; Q1, 25th percentile; Q3, 75th percentile.

	Frequency (N = 50)	Percent
Sex		
Female	7	14%
Male	43	86%
Surgery Performed		
Craniectomy	26	52%
Craniotomy	24	48%
Type of injury		
Closed head injury	40	80%
Skull fracture	8	16%
Gunshot wound	2	4%
Complications		
CNS infection (RTO)	3	6%
CSF Leak (RTO)	1	2%
Rebleed (RTO)	2	4%
None	44	88%
Mortality		
Death	6	12%
Survived	44	88%
Presenting GCS		
GCS 3 to 5	23	46%
GCS 6 to 8	27	54%
Effaced Cisterns	22	44%
Disproportional MLS	12	24%
Glasgow Outcome Score (GOS)		
1	6	12%
2	5	10%
3	13	26%
4	10	20%
5	16	32%
Age (years)	22.38 ± 3.12	
Hospital Length of Stay, median (Q1, Q3)	13.5 (7,30)	

When comparing patients selected for craniotomies to patients selected for craniectomies; patients who underwent a craniotomy were more likely to have a presenting GCS score between 6 and 8 (70.8% vs 38.5%, p = 0.0218), a higher postoperative chance of survival (100% vs 76.9%, p = 0.0121), a more favorable outcome (87.5% vs 19.2%, p < 0.0001), a lower incidence of effaced basal cisterns (12.5% vs 73.1%, p < 0.001), a lower incidence of disproportional MLS (4.2% vs 46.1% p = 0.00036) and a shorter median length of stay (11 days vs 23 days, p = 0.0008). Of the five patients from the craniectomy group with a favorable outcome, all had a presenting GCS of 6-8 (Table 2).

**Table 2:** Cross-tabulation analysis of factors associated with the two surgical procedures Abbreviations: CNS, central nervous system; CSF, cerebrospinal fluid; GCS, Glasgow Coma Scale; RTO, Return to operating room; Q1, 25th percentile Q3, 75th percentile.

	Craniectomy (n=26)	Craniotomy (n=24)	P-value
GCS at time of presentation			0.0218
GCS 3 to 5	16 (61.5%)	7 (29.2%)	
GCS 6 to 8	10 (38.5%)	17 (70.8%)	
Effaced Cisterns	19 (73.1%)	3 (12.5%)	< 0.0001
Midline shift out of proportion	12 (46.1%)	0 (0%)	0.00036
Glasgow Outcome Score			< 0.0001
Unfavorable - Glasgow Outcome Score (1 through 3)	21 (80.7%)	3 (12.5%)	
Favorable - Glasgow Outcome Score (4 through 5)	5 (19.2%)	21 (87.5%)	
Mortality			0.0121
Death	6 (23.1%)	0 (0%)	
Survived	20 (76.9%)	24 (100%)	
Complications			0.1015
CNS infection (RTO)	3 (11.5%)	0 (0%)	
CSF Leak (RTO)	0 (0%)	1 (4.2%)	
Rebleed (RTO)	2 (7.7%)	0 (0%)	
None	21 (80.8%)	23 (95.8%)	
Mean Age	22.65 ± 3.01	22.08 ± 3.28	0.5242
Hospital Length of Stay, Median (Q1, Q3)	23 (11,41)	11(5, 14.5)	0.0008

All six mortalities occurred in patients presenting with a GCS of 3-5. These patients all had effaced basal cisterns, disproportional MLS, and underwent a craniectomy.

Twelve patients in the entire cohort were found to have disproportional MLS; of these 12 patients, all suffered a CHI. DSFs were observed in eight patients; all of which survived, and only one of these eight had effaced basal cisterns and an unfavorable 30-day outcome (GOS 2).

A logistic regression analysis identified that patients selected for a craniectomy with a GCS of 3-5 were 19.06 times more likely to have unfavorable clinical outcomes (Table 3). Moreover, patients who presented with a GCS score from 3 to 5 (in both groups) were 4.99 times more likely to have unfavorable clinical outcomes.

**Table 3:** Predictors of unfavorable clinical outcomes after surgery (defined as Glasgow Outcome Score 1 through 3) Abbreviations: CI, confidence interval; GCS, Glasgow Coma Scale; OR, odds ratio

	Unadjusted OR with 95% CI	Adjusted OR with 95% CI
Surgery		
Craniotomy	Reference	Reference
Craniectomy	21.11 (4.61, 96.67)	19.06 (3.79, 96)
Presenting GCS		
Presenting GCS 6 to 8	Reference	Reference
Presenting GCS 3 to 5	5.88 (1.69, 20.42)	4.99 (1.05, 23.74)
Age	Not significant	Not significant
Gender	Not significant	Not significant
Mechanism of injury	Not significant	Not significant

Predictors of unfavorable outcome stratified based on presenting GCS are listed in Table 4 and Table 5. Of the patients GCS 3-5 upon presentation (N = 23); 7 underwent a craniotomy and 16 underwent a craniectomy. Of the 16 that underwent a craniectomy, all had an unfavorable outcome and 14 had effaced basal cisterns (Table 4). Seven patients in the GCS 3-5 group had a favorable outcome; of these seven, all had open basal cisterns, none had disproportional MLS and 4 had a depressed skull fracture (Table 4). Conversely, when looking at patients GCS 6-8 upon presentation (N = 27), 17 underwent a craniotomy and 10 underwent a craniectomy. Of the 10 that underwent a craniectomy, five had an unfavorable outcome (Table 5).

**Table 4:** Predictors of unfavorable clinical outcomes after surgery (defined as Glasgow Outcome Score 1 through 3) in patients presenting as GCS 3-5. Craniotomy N = 7, Craniectomy N = 16 Abbreviations: GCS, Glasgow coma scale; MLS, Mid Line Shift; SOL, Space Occupying Lesion; TOI, Type of Injury severe TBI; CHI, Closed Head Injury; DSF, Depressed Skull fracture; Unfavorable + = outcome was death

GCS	Surgery	MLS > SOL	Cisterns	TOI	Outcome
3	Craniotomy	No	Open	DSF	Favorable
3	Craniotomy	No	Open	DSF	Favorable
3	Craniotomy	No	Open	DSF	Favorable
3	Craniotomy	No	Open	CHI	Favorable
3	Craniotomy	No	Open	CHI	Favorable
3	Craniectomy	No	Effaced	CHI	Unfavorable
3	Craniectomy	No	Effaced	CHI	Unfavorable
3	Craniectomy	Yes	Open	CHI	Unfavorable
3	Craniectomy	No	Effaced	CHI	Unfavorable
3	Craniectomy	No	Effaced	CHI	Unfavorable
3	Craniectomy	Yes	Effaced	CHI	Unfavorable +
3	Craniectomy	Yes	Effaced	CHI	Unfavorable +
4	Craniotomy	No	Open	DSF	Favorable
4	Craniotomy	No	Open	CHI	Unfavorable
4	Craniectomy	No	Effaced	CHI	Unfavorable
4	Craniectomy	No	Effaced	DSF	Unfavorable
4	Craniectomy	Yes	Effaced	CHI	Unfavorable
4	Craniectomy	Yes	Effaced	CHI	Unfavorable
4	Craniectomy	Yes	Effaced	CHI	Unfavorable +
4	Craniectomy	Yes	Effaced	CHI	Unfavorable +
4	Craniectomy	Yes	Effaced	CHI	Unfavorable +
5	Craniectomy	No	Open	CHI	Favorable
5	Craniectomy	Yes	Effaced	CHI	Unfavorable +

**Table 5:** Predictors of unfavorable clinical outcomes after surgery (defined as Glasgow Outcome Score 1 through 3) in patients presenting as GCS 6-8. Craniotomy N = 17, Craniectomy N = 10 Abbreviations: GCS, Glasgow coma scale; MLS, Mid Line Shift; SOL, Space Occupying Lesion; TOI, Type of Injury severe TBI; CHI, Closed Head Injury; DSF, Depressed Skull fracture

GCS	Surgery	MLS > SOL	Cisterns	TOI	Outcome
6	Craniectomy	No	Open	CHI	Favorable
6	Craniotomy	No	Open	CHI	Favorable
6	Craniotomy	No	Open	DSF	Favorable
6	Craniotomy	No	Open	DSF	Favorable
6	Craniotomy	No	Open	CHI	Favorable
6	Craniectomy	Yes	Effaced	CHI	Unfavorable
6	Craniotomy	No	Effaced	CHI	Unfavorable
6	Craniectomy	No	Effaced	CHI	Unfavorable
7	Craniotomy	No	Open	CHI	Favorable
7	Craniotomy	No	Open	CHI	Favorable
7	Craniotomy	No	Open	CHI	Favorable
7	Craniotomy	No	Effaced	GSW	Favorable
7	Craniotomy	No	Open	CHI	Favorable
7	Craniotomy	No	Open	CHI	Favorable
7	Craniotomy	No	Open	GSW	Favorable
7	Craniotomy	No	Open	DSF	Favorable
7	Craniotomy	No	Open	CHI	Favorable
7	Craniectomy	Yes	Open	CHI	Favorable
7	Craniotomy	No	Effaced	CHI	Favorable
7	Craniectomy	No	Open	CHI	Favorable
7	Craniotomy	No	Open	CHI	Favorable
7	Craniectomy	No	Effaced	CHI	Favorable
7	Craniectomy	No	Open	CHI	Unfavorable
7	Craniectomy	No	Open	CHI	Unfavorable
7	Craniectomy	Yes	Effaced	CHI	Unfavorable
8	Craniectomy	No	Effaced	CHI	Favorable
8	Craniotomy	No	Open	CHI	Favorable

Review of postoperative CT scans revealed that all patients with unfavorable outcomes had persistent effaced cisterns, persistent MLS > SOL or both.

## Discussion

Our retrospective data analysis of young adults requiring emergent decompression after severe TBI offers foresight into overall outcome of this patient population. A young and healthy adult population serves as an adequate model to assess functional recovery after severe TBI as this population is generally absent of confounding co-morbid conditions seen in older patients [18-20].

Factors such as disproportional midline shift, GCS 3-5 on arrival, closed head injury, and effaced basal cisterns all had a statistically significant impact on probability of survival and outcome. All patients in the cohort with GCS 3-5 on presentation, effaced basal cisterns, disproportional midline shift, and closed head injury had an unfavorable outcome, 6 resulting in mortality.

Persistent effacement of basal cisterns, persistent disproportional MLS, or both, within the initial 24 hours of severe TBI were associated with all patients (GCS 3-5 and GCS 6-8 groups) that had a short-term unfavorable outcome at 30 days. This suggests that only relying on an initial 10 cm x 15 cm craniectomy may not be sufficient to relieve the disproportional MLS or effaced basal cisterns in patients that have deteriorated to a GCS 3-5 and in some patients with a GCS 6-8. All patients in the GCS 6-8 group with effaced cisterns or disproportional MLS with a favorable outcome had improved MLS and open basal cisterns observed on postoperative CT scans. This suggests that there is a degree of compressive effect on the brainstem or blood vessels during a period of increased metabolic demand in severe TBI patients with a GCS 6-8 that can be quickly relieved for a favorable outcome. Perhaps future studies emphasizing a technique of immediate maximal decompression of the middle fossa to adequately relieve the shift and effaced cisterns in patients with GCS 3-5 and restore blood flow can add insight into the surgical treatment of this pathological condition.

When comparing closed head injuries (CHI) to depressed skull fractures (DSF), patients with CHIs had worse outcomes and were more likely to undergo a craniectomy for adequate decompression. Moreover, no patients with DSFs had disproportional MLS and all but one of these patients had a favorable outcome. We hypothesize that open skull fractures and dural injury with cerebrospinal fluid (CSF) leak may provide interim relief of intracranial hypertension by allowing atmospheric pressure to influence intracranial pressure and CSF diversion.

In our cohort, all patients with effaced basal cisterns, disproportional MLS>SOL, and CHI had an unfavorable outcome regardless of presenting GCS or surgery performed. Moreover, all the mortalities occurred in patients presenting with GCS 3-5, effaced basal cisterns, MLS>SOL and CHI. GCS 3-5 upon presentation overall had worse outcomes when compared to GCS 6-8 upon presentation. This suggests that patients GCS 3-5 upon presentation, with effaced basal cisterns, and MLS>SOL still have an unfavorable 30-day outcome after an initial 10 cm X 15 cm decompressive craniectomy. It also suggests that patients presenting with all the aforementioned findings are more likely to have a 30-day mortality worse than those without the aforementioned findings.

This study is not without limitations. The retrospective nature of the study is a limitation, and the demographics of the study (patients age 18 to 30 years) preclude correlating this data with the general adult population. The small sample size is another limitation; however, the inclusion and exclusion criteria allow us to focus our attention on a patient population likely to suffer from severe TBI without conflicting effects from comorbid conditions. The study does not include patients with isolated epidural hematomas without associated parenchymal injury; however, the reason for exclusion is because these patients typically have a better prognosis after surgical decompression [15-16]. Thirty-day follow up is a limitation and the authors are aware that functional recovery occurs over months to years. We hope that future studies may include a multi-centered analysis with long-term follow-up.

## Conclusions

Severe TBI is a devastating ailment seen in young adults. Factors found to pertain to poor outcome in our cohort include GCS 3-5 on presentation, effaced basal cisterns, disproportional MLS>SOL, and closed head injury. In this study, the association continues if the conditions persist after a 10 cm X 15 cm initial decompressive craniectomy. Patients undergoing a craniectomy overall had an unfavorable outcome when compared to patients undergoing a craniotomy; however, we believe this may be secondary to a more severe underlying pathological process that was neither qualified or quantified and was not relieved with the initial craniectomy. As would be expected, young adult patients suffering from severe TBI with GCS 3-5 on presentation, MLS > SOL, effaced basal cisterns, and CHI are more likely to have an unfavorable outcome or mortality when compared to patients with GCS 6-8 on presentation, effaced basal cisterns, and disproportional MLS even if treated with a decompressive craniectomy upon arrival. These physical and radiographic findings can offer insight into the overall disease process of severe TBI and can be used to educate and inform family members of patients with severe TBI. We hope that this retrospective review leads to further investigation regarding predictive factors of overall outcome in this patient population.
